# Knowledge and perceptions about schistosomiasis among primary school children and teachers in rural KwaZulu-Natal

**DOI:** 10.4102/sajid.v35i1.126

**Published:** 2020-07-06

**Authors:** Edmore Mazani, Myra Taylor, Eyrun F. Kjetland, Patricia D. Ndhlovu

**Affiliations:** 1 Department of Public Health Medicine, Faculty of Health Sciences, University of KwaZulu-Natal, Durban, South Africa; 2 Department of Infectious Diseases, Norwegian Centre for Imported and Tropical Diseases, Oslo University Hospital, Oslo, Norway; 3 Department of Infectious Diseases, Faculty of Health Sciences, Imperial College London, London, United Kingdom

**Keywords:** urinary schistosomiasis, knowledge, perceptions, mass drug administration, praziquantel

## Abstract

**Background:**

Schistosomiasis is a disease caused by parasitic trematode worms of the genus Schistosoma. In 2014, over 258 million people worldwide required treatment for the disease. Schistosomiasis is known to be prevalent in the northern region of KwaZulu-Natal province of South Africa, especially among school-going children but less is known about their knowledge of the disease and their attitude towards being treated for the disease at school.

**Methods:**

The study was a descriptive and analytical cross-sectional survey conducted through self-administered questionnaires among grades 5 and 7 learners from 10 randomly selected rural primary schools in iLembe and uThungulu, KwaZulu-Natal. Teachers from the same schools participated during the same period.

**Results:**

A total of 730 learners and 78 teachers took part in the study. Among the learners, 73.2% (95% confidence interval [CI]: 69.7% – 76.4%) correctly identified freshwater contact as a risk for schistosomiasis, but only 42.7% (95% CI: 38.8% – 46.8%) knew how to prevent it. Among the teachers, 96.8% (95% CI: 87.8% – 99.4%) knew the risk and 69.0% (95% CI: 55.3%– 80.1%) knew the prevention of schistosomiasis. Almost 70% (95% CI: 65.9% – 72.8%) of the learners and 67.6% (95% CI: 42.1% – 65.6%) of the teachers reported their willingness to receive treatment with praziquantel at school.

**Conclusion:**

This study showed that basic knowledge about the risk of schistosomiasis among the participants was high, but the cause and prevention of the disease were less well understood. There is need to include schistosomiasis in health education both at school and through community awareness programmes.

## Introduction

Schistosomiasis (also called bilharzia) is a disease caused by parasitic trematode worms (schistosomes) of the genus *Schistosoma*. More than 258 million people worldwide required treatment for schistosomiasis in 2014.^1^ Of these, more than 90% live in Africa.^2^ In South Africa (SA), it is estimated that 5.2 million people need treatment for schistosomiasis and KwaZulu-Natal (KZN) province is one of the worst affected areas with a schistosomiasis prevalence of between 22% and 55% in children.^1,2^ Affected children may suffer from poor growth and impaired cognitive development.^3^ Urinary schistosomiasis may also cause haematuria, pain during urination (dysuria), lesions in the bladder, anaemia, malnutrition, lethargy and longer term kidney failure, leading to an increased risk of bladder cancer and reproductive tract morbidity that may lead to infertility.^4^ Women may also suffer from female genital schistosomiasis (FGS) and thus be at risk of contracting human immunodeficiency virus (HIV).^4^ Schistosomiasis infection disrupts school attendance and classroom attention, along with a range of social, psychological and long-term economic consequences.^4,5,6,7,8^ The prevalence and intensity of infection peak in the 10- to 14-year age group.^5^

To prevent morbidity in endemic areas, the World Health Organization (WHO) recommends mass drug administration (MDA) with praziquantel (PZQ) targeting not only school children, but also adults at risk of schistosomiasis.^5^ Treatment if given several times during childhood would likely prevent the adulthood long-term consequences of the morbidity.^5^ In 2012, WHO developed a policy document with the goal to eliminate schistosomiasis as a public health problem by 2025. In line with the WHO goal, millions of school-aged children living in endemic areas are to receive free PZQ drugs through international supplied PZQ for use in MDA campaigns.^8^ In countries outside SA, teachers play a very important role in MDA campaigns and in increasing awareness about the disease,^9,10^ something that can potentially be adopted in SA.

The WHO recommends that at least 75% of school children in endemic areas should receive treatment in order for MDA to be effective in controlling.^5,8^ Such compliance requires excellent community liaison. In addition, more than just the treatment is required.^9,10^ Increasing access to safe clean water, adequate sanitation and behaviour change may also be effective ways to prevent the disease.^10,11,12^ Hence, health education and health promotion campaigns are very important. The targeted population must be clearly identified and a clear message concerning treatment should be delivered.^10,11^ This will be easier if local knowledge and perceptions are taken into account. Schistosomiasis is known to be prevalent in the northern region of KZN province, especially among school-going children but less is known about their knowledge of the disease and their attitude towards being treated for the disease at school.^13,14^ The information may also help develop policies to control and treat schistosomiasis in schools. Thus, in this study, we explore the knowledge of schistosomiasis among teachers and learners and investigate their attitude to MDA at school. While major factors may affect the ability by children to understand the implications of behaviour on one’s health, this study does not delve deeper into that.

## Methodology

### Study area and population

The study was conducted among rural learners and teachers attending KZN Department of Basic Education schools. The rural populations in iLembe and uThungulu are mainly Zulu speaking and agriculture is their primary economic activity. These districts were selected as schistosomiasis endemic areas in the province of KZN.^13^

Ten rural primary schools in Lower Tugela Circuit of Ilembe district and Samungu Circuit of uThungulu district that are schistosomiasis endemic areas were randomly selected from the KZN Department of Education school lists. Five schools from each district were selected. This area contains the Tugela River and many smaller rivers and streams that are all used for domestic, recreational and agricultural purposes. Schools that took part or are taking part in any schistosomiasis research currently taking place at the University of KwaZulu-Natal were excluded from the study.

All grade 5 and 7 learners were invited to participate. This group was chosen because their ages ranged from 10 to 14 years, which is the age group carrying the highest burden of the schistosomiasis in endemic areas.^5,14^ All teachers in the selected schools were also invited to participate.

### Study sample

The sample was calculated using the formula: $N=\frac{Z^{2}P(1-P)}{d^{2}}$ as described by Lwanga and Lemeshow.^22^ A sample size of 385 was established using the following parameters: *Z*-statistic is 1.96 for the confidence level of 95%; *P* is 0.5 as the expected proportion of the characteristic to be measured in the study area^15^; *d* is the precision of 0.05 for the 95% confidence interval (CI). To allow for any intraclass correlation between schools and increase the chances of having enough participants even when others would not consent to participation, more participants than the calculated sample size were invited to participate.

### Questionnaire survey

The study was a descriptive and analytical cross-sectional survey conducted through self-administered, previously piloted questionnaires. The piloting was performed in a school not participating in the study. No participant put his or her name to the coded questionnaire. All participants filled their questionnaires without the assistance of their colleagues to ensure that they would answer as truthfully as possible. The questionnaires were translated into IsiZulu, back-translated into English for clarity, pretested and adapted to local decorum. The data were collected during April and June 2017.

For open-ended questions, answers were classified as correct or incorrect. Answers were considered correct if they included information on contact with faecally or urine-polluted water, freshwater (river, stream or dam) contact, swimming in freshwater and playing in freshwater, the use of water that is not treated and improper human excreta disposal. In relation to prevention, answers were considered correct if they mentioned proper excreta disposal and avoiding contact with freshwater sources. Incorrect answers were those that had nothing to do etiologically with schistosomiasis.

### Data analysis

Data collected were entered in Excel and analysed using SPSS 24.0 software (IBM Corp., Armonk, NY, USA). The standard Pearson’s χ^2^ and odds ratio (OR) with 95% CIs were used to determine associations between various variables. Multivariable logistic regression analysis was used to ascertain the association between selected predictors and outcome variables. The outcome variables were knowledge of transmission of schistosomiasis and willingness to accept schistosomiasis morbidity-preventative treatment at school. Associated variables with a *p*-value of less than 0.20 were included in the multivariable regression analysis. A significance level of 5% was used.

### Ethical consideration

Ethical clearance for the study was obtained from the Humanities and Social Sciences Research Ethics Committee of the University of KwaZulu-Natal (Protocol Reference Number: 0404/016M). Permission and approval to conduct the research was granted by the KwaZulu-Natal Department of Education (Reference number: 2/4/8/877). Permission was also obtained from the school principals and parents before the study was conducted. Information about the study was translated into isiZulu and sent home to parents or caregivers and written informed consent was requested. All learners provided assent.

## Results

### General characteristics of the study population

#### Learners

A total of 730 learners from 10 primary schools took part in this study. Of the learners, 396 (54.2%) were girls, while 334 (45.8%) were boys. Four-hundred and fourteen (56.7%) of the learners were in grade 5 and 316 (43.3%) in grade 7. The ages of learners were normally distributed. The mean age of all the learners was 12.04 years (SD 1.569) and the ages ranged from 9 to 18 years. The mean age of women in grade 5 was 10.82 years (SD 0.918) and the mean age of female learners in grade 7 was 12.92 years (SD 1.047). The mean age of male learners in grade 5 was 11.54 years (SD 1.217) and the mean age of male learners in grade 7 was 13.23 years (SD 1.252).

Among the learners, 251 out of 711, 35.3% (95% CI: 31.8% – 39.0%), reported having had schistosomiasis; 255 out of 720, 35.4% (95% CI: 31.9% – 39.1%), learners reported having had traces of blood in urine and 272 out of 705, 38.6% (95% CI: 35.0% – 42.3%), reported having had pain during urination. About one-fifth (133/657, 20.2%; 95% CI: 17.3% – 23.6%) learners reported having received some treatment for schistosomiasis and 55 out of 657, 8.4% (95% CI: 6.42% – 10.8%), of these reported having received a form of traditional treatment for schistosomiasis. Twenty-eight out of 647 (4.33%; 95% CI: 2.95% – 6.28%) had received treatment both the clinically and traditionally. About a third (259/722, 35.9%; 95% CI: 32.4% – 39.5%) of the learners were taught about schistosomiasis at home and 214 out of 715, 29.9% (95% CI: 26.6% – 33.5%), reported having been taught at school. There was little clustering effect between the 10 schools. For example, the intraclass correlation coefficient for treatment for schistosomiasis was 0.01 (95% CI: 0.067–0.086).

#### Teachers

Seventy-eight teachers participated in the study. The median age of the teachers was 41 years with their ages ranging from 23 to 62 years. Of the 78 teachers, 59 (75.6%) were female and 19 (24.4%) were male. About half (40/76, 52.6%; 95% CI: 40.9% – 64.1%) of the teachers reported that schistosomiasis was prevalent in their area. Twenty-four out of 77 (31.2%; 95% CI: 21.4% – 42.9%) of the teachers reported having themselves had schistosomiasis. Among the teachers, 23 out of 78, 29.5% (95% CI: 20.0% – 41.1%), reported having had red urine and 29 out of 78, 37.2% (95% CI: 26.7% – 48.9%), reported having had pain during urination. Eighteen teachers out of 77 (23.4%; 95% CI: 14.8% – 34.7%) had been treated for schistosomiasis, and 17 of whom were treated at a clinic and one traditionally. Almost half of the teachers (41/77, 53.2%; 95% CI: 41.6% – 64.6%) reported having been taught about schistosomiasis at school. Thirty-eight out of 74 (51.4%; 95% CI: 39.5% – 63.0%) of the teachers reported that they had been taught at home about schistosomiasis.

### Knowledge of transmission and prevention of schistosomiasis

Table 1 shows the responses that were given by learner participants to questions relating to acquisition and prevention of schistosomiasis. Correct responses on how people get and prevent schistosomiasis were significantly associated with the grade of a learner (adj OR: 1.812; 95% CI: 1.253–2.622; *p* = 0.002) and reported having had schistosomiasis previously (adj OR: 1.780; 95% CI: 1.180–2.687; *p* = 0.006).

**TABLE 1:** Learners’ responses to questions on infection and prevention of schistosomiasis by grade, sex and district.

Question	Responses given by respondents	Grade 5		Grade 7		Female		Male		Overall	
		%	95% CI	%	95% CI	%	95% CI	%	95% CI	%	95% CI
How do people get Bilharzia?	Swimming or playing in rivers or dirty water	68.2	63.3–72.7	79.6	74.6–83.9	69.2	64.3–73.7	78.0	73.0–82.4	73.2	69.7–76.4
	Having blood in urine	3.05	1.66–5.41	0.99	0.26–3.10	2.35	1.15–4.57	1.91	0.78–4.32	2.15	1.25–3.61
	Having sexual intercourse with an infected person	0.00	0.00–1.21	1.32	0.42–3.34	0.52	0.09–2.08	0.64	0.11–2.54	0.57	0.18–1.56
	Jumping the fire	1.01	0.33–2.77	0.66	0.11–2.62	0.00	0.00–1.24	1.91	0.78–4.32	0.86	0.35–1.96
	I don’t know	17.3	13.8–21.5	12.8	9.39–17.2	20.9	17.0–25.4	8.60	5.85–12.4	15.4	12.8–18.3
	Other	10.4	7.67–14.0	4.61	2.64–7.79	7.05	4.78–10.2	8.82	6.11–12.77	7.89	6.05–10.2
How can people prevent Bilharzia infection?	Avoid swimming in dirty water or rivers	33.8	28.8–39.2	53.6	47.8–59.6	39.3	34.1–44.7	47.1	41.0–53.1	42.7	38.8–46.8
	Going to clinic or doctors or tablets	24.0	19.7–29.0	18.5	14.2–23.7	22.9	18.6–27.8	19.9	15.38–25.2	21.5	18.4–25.0
	Avoid sexual intercourse with an infected person	0.89	0.23–0.28	0.72	0.12–2.87	1.17	0.37–3.18	0.37	0.02–2.36	0.82	0.30–2.01
	Avoid contact with an infected person	4.45	2.60–7.39	2.54	1.12–5.38	3.52	1.92–6.23	3.68	1.88–6.87	3.59	2.32–5.47
	Going to the traditional healer	0.59	0.10–2.36	0.36	0.02–2.32	0.88	0.23–2.77	0.00	0.00–1.74	0.16	0.13–1.55
	I don’t know	24.3	19.9–29.3	16.3	12.3–21.3	25.2	20.8–30.3	15.1	11.2–20.0	20.7	17.6–24.2
	Other	11.9	8.71–15.9	7.97	5.18–11.98	7.04	4.66–10.4	14.0	10.2–18.8	10.1	7.89–12.8

CI, confidence interval.

All the teachers associated dirty water contact with schistosomiasis infection; however, 96.8% answered correctly on how people get schistosomiasis. A few (3/58, 5.17%; 95% CI: 1.34% – 15.3%) teachers reported that they do not know how to prevent schistosomiasis.

### Perceptions about schistosomiasis

The perceived harm and embarrassment associated with schistosomiasis is reported in Tables 2 and 3. Table 2 shows that male learners were twice as likely to perceive schistosomiasis as harmful compared with female learners (adj OR: 2.065; 95% CI: 1.446–2.944; *p* < 0.001).

**TABLE 2:** Pearson’s χ^2^ test and multivariable logistic regression analyses of factors affecting the perception of schistosomiasis as harmful by learners.

General description		Perceive schistosomiasis as harmful %		Pearson’s *χ*^2^ test		Multivariable analysis		
Factor	Variable	%	95% CI	*χ* ^2^	*p* *	Adjusted OR	95% CI	*p* *
Grade	-	-	-	2.273	0.132	-	-	-
	5	70.0	65.2–74.3	-	-	1	-	-
	7	64.6	59.1–69.9	-	-	0.720	0.516–1.006	0.054
Sex	-	-	-	19.811	<0.001	-	-	-
	Female	60.6	55.5–65.4	-	-	1	-	-
	Male	75.1	71.1–80.6	-	-	2.065	1.446–2.944	< 0.001
District†	-	-	-	0.421	0.517	-	-	-
	iLembe	66.7	61.9–71.2	-	-	-	-	-
	uThungulu	69.0	63.4–74.0	-	-	-	-	-
Have had schistosomiasis	-	-	-	4.523	0.034	-	-	-
	No	65.3	60.7–69.6	-	-	1	-	-
	Yes	73.1	67.1–78.4	-	-	1.111	0.768–1.608	0.575
Have been taught about schistosomiasis at school	-	-	-	3.242	0.072	-	-	-
	No	65.4	61.0–69.5	-	-	1	-	-
	Yes	73.7	67.2–79.4	-	-	1.085	0.742–1.587	0.674
Have been taught about schistosomiasis at home	-	-	-	21.170	< 0.001	-	-	-
	No	61.7	57.0–66.1	-	-	1	-	-
	Yes	78.4	72.8–83.1	-	-	2.520	1.721–3.692	< 0.001

* , *p* < 0.05 is the level of statistical significance.

† , Not included in the multivariable analysis because *p* > 0.2.

**TABLE 3:** Pearson’s χ^2^ test and multivariable logistic regression analyses of factors affecting whether a learner would disclose to their friend were they to have schistosomiasis.

General description		Would disclose to a friend were they to be diagnosed with schistosomiasis		Pearson’s *χ*^2^ test		Multivariable analysis		
Factor	Variable	%	95% CI	*χ* ^2^	*p* *	Adjusted OR	95% CI	*p* *
Grade†	-	-	-	0.172	0.678	-	-	-
	5	39.0	34.3–44.1	-	-	-	-	-
	7	40.6	35.1–46.3	-	-	-	-	-
Sex	-	-	-	19.973	< 0.001	-	-	-
	Female	32.2	27.6–37.2	-	-	1	-	-
	Male	48.8	43.2–54.4	-	-	1.714	1.234–2.380	0.001
District†	-	-	-	0.554	0.457	-	-	-
	ILembe	40.9	36.1–45.8	-	-	-	-	-
	uThungulu	38.1	32.6–43.9	-	-	-	-	-
Have had schistosomiasis	-	-	-	12.127	0.001	-	-	-
	No	34.8	30.4–39.4	-	-	1	-	-
	Yes	48.3	41.9–54.8	-	-	1.460	1.051–2.030	0.021
Have been taught about schistosomiasis at school	-	-	-	5.109	0.024	-	-	-
	No	37.0	32.7–41.5	-	-	1	-	-
	Yes	46.2	39.3–53.2	-	-	1.442	1.029–2.022	0.034
Have been taught about schistosomiasis at home†	-	-	-	0.395	0.530	-	-	-
	No	38.9	34.5–43.6	-	-	-	-	-
	Yes	41.4	35.2–47.8	-	-	-	-	-

* , *p* < 0.05 is the level of statistical significance.

† , Value not included in the multivariable analysis because *p* > 0.2.

More male learners (156/320, 48.8%; 95% CI: 43.2% – 54.4%) reported that they would disclose to their friends were they to be diagnosed with schistosomiasis compared to female learners (124/385, 32.2%; 95% CI: 27.6% – 37.2%) (Table 3).

More than half of the teachers (48/75, 64.0%; 95% CI: 52.0% – 74.5%) believed that schistosomiasis affects a learner’s ability to learn, while 39 out of 73 (53.4%) (95% CI: 41.4% – 65.0%) believed that schistosomiasis could affect a person’s sex life.

### Willingness to take schistosomiasis treatment at school

As shown in Table 4, the willingness to take schistosomiasis treatment at school by learners was significantly associated with having had pain during urination (adj OR: 2.923; 95% CI: 1.848–4.624; *p* < 0.001), and the perception of schistosomiasis as harmful (adj OR: 1.937; 95% CI: 1.331–2.820; *p* = 0.001).

**TABLE 4:** Pearson’s χ^2^ test of factors affecting the willingness of learners and teachers to receive treatment at school.

General description		Pearson’s *χ*^2^ test				Multivariable analysis		
Factor	Variable	Would accept treatment at school		*χ* ^2^	*p* *	Adjusted OR	95% CI	*p* *
		%	95% CI					
Grade	-	-	-	3.877	0.049	-	-	-
	5	72.5	67.7–76.8	-	-	1	-	-
	7	65.6	60.0–70.8	-	-	0.707	0.492–0.061	0.061
Sex		-	-	1.335	0.248	-	-	-
	Female	67.6	62.6–72.2	-	-	-	-	-
	Male	71.7	66.3–76.5	-	-	-	-	-
District	-	-	-	2.173	0.141	-	-	-
	iLembe	67.3	62.5–71.8	-	-	1.220	0.846–1.758	0.287
	uThungulu	72.5	66.9–77.5	-	-	1	-	-
Have had schistosomiasis	-	-	-	18.950	< 0.001	-	-	-
	No	64.6	59.9–69.0	-	-	1	-	-
	Yes	80.5	74.8–85.2	-	-	1.717	0.938–3.144	0.080
Taught at school about schistosomiasis	-	-	-	0.055	0.814	-	-	-
	No	70.4	66.1–74.4	-	-	-	-	-
	Yes	69.3	62.4–75.4	-	-	-	-	-
Taught at home about schistosomiasis	-	-	-	0.233	0.629	-	-	-
	No	68.8	64.3–73.0	-	-	-	-	-
	Yes	70.6	64.4–76.1	-	-	-	-	-
Perceive schistosomiasis as harmful	-	-	-	16.882	< 0.001	-	-	-
	No	59.3	52.6–65.7	-	-	1	-	-
	Yes	74.6	70.4–78.4	-	-	1.937	1.331–2.820	0.001
Have had red urine	-	-	-	15.462	< 0.001	-	-	-
	No	64.5	59.9–68.9	-	-	1	-	-
	Yes	78.9	73.1–83.7	-	-	0.784	0.417–1.471	0.448
Have had pain during urination	-	-	-	39.575	< 0.001	-	-	-
	No	60.8	56.0–65.5	-	-	1	-	-
	Yes	83.3	78.2–87.5	-	-	2.923	1.848–4.624	< 0.001
Knew how people get schistosomiasis	-	-	-	1.821	0.178	-	-	-
	No	65.7	58.3–72.5	-	-	1	-	-
	Yes	71.1	66.9–75.1	-	-	0.973	0.643– 1.475	0.899

CI, confidence interval.

* , *p* < 0.05 is the level of statistical significance; variables *p* < 0.2 were not included in regression analysis.

Out of 74, 50 (67.6%; 95% CI: 55.6% – 77.7%) teachers reported that they would take schistosomiasis tablets were they to be brought to their schools by nurses. Furthermore, 44 out of 74 (59.5%; 95% CI: 47.4% – 70.5%) said that they were willing to be checked by doctors for schistosomiasis.

## Discussion

The fact that more than 70% of the learners answered correctly as to how people get schistosomiasis shows that the learners had prior education about schistosomiasis. These findings were similar to the study that was conducted in Lowveld Swaziland in 2014 among grades 5–7.^16^ That study found that 74% of the learners correctly identified dams and rivers as a risk factor for getting schistosomiasis. A study conducted in Zimbabwe in 2004 among grade 3 learners provided different findings.^17^ In that study, only 32% of the learner participants knew about the risks of swimming in rivers or dams for schistosomiasis, which is lower than in this study, but learners were older being in grades 5 and 7.

In South African schools, schistosomiasis is not part of the formal syllabus in both primary and secondary schools.^18^ However, this study showed that some schools offer some education on schistosomiasis. About 30% of the learners who participated reported having been taught about schistosomiasis at school. This is commendable, but to control the disease successfully, more formalised and targeted awareness should be considered in schools. It was also interesting to note from this study that more than a quarter of the learners reported that they were taught at home about schistosomiasis. It is evident from the results that not only home and school are the sources of bilharzia information, as shown by the over 701% of the learners who correctly knew how people get schistosomiasis, suggesting informal communication among learners. The content of the tuition that participants had received about schistosomiasis and the potential role of the clinic were not explored further in this study.

Many learners and teachers were aware that by swimming in freshwater areas, they could get *isichenene* (isiZulu word for schistosomiasis) but the understanding of the reasons for this appeared to be less well understood. Other studies have also found that many people perceive schistosomiasis as a water-borne infection that is transmitted through contact with freshwater sources, but they fail to describe the role of snails in the life cycle of the disease.^16,17,23^

The importance of improving sanitation and ensuring hygienic urination and defecation is necessary for the long-term management of this disease.^21^ It was found in this study that all participants (both learners and teachers) did not mention improper disposal of human excreta in both the transmission and prevention of schistosomiasis. This could be because they did not understand well the role of improper disposal of human excreta in the life cycle of schistosomiasis.

It was interesting to note that the perceived harm of schistosomiasis was significantly associated with the sex of the participant, with more male learners perceiving schistosomiasis as harmful compared with females. This association is worrying because studies have shown that as children grow into their late teens, more girls than boys suffer from schistosomiasis.^20^ A change in perception is needed if fewer girls are to be infected with schistosomiasis. Girls need to be aware of the dangerous effects of schistosomiasis particularly in respect of FGS, and the possible association of schistosomiasis with HIV and also possible infertility, to name a few of the possible consequences of the infection.^6,7^

This study found that about two-thirds (489/704, 69.5%; 95% CI: 65.9% – 72.8%) of the learners who participated in the study would take the treatment tablets were a nurse to bring them to their schools. A study conducted in Ugu District, KZN, found that on average 44% of the learners in both primary and secondary schools took the offered treatment (MDA) at their schools.^15^ The study in Ugu found that 50% of learners in primary school took the treatment. The lower percentage in Ugu could be because some of the learners lost their consent forms or did not have an adult at home to sign their consent form.^15^

Among the teachers, 40 out of 74, 67.6% (95% CI: 55.6% – 77.7%), reported that they would accept treatment for schistosomiasis at school. Possible reasons for this low percentage could be that teachers view schistosomiasis as a disease for children and believe that they themselves are not at risk of the disease. However, their views could potentially negatively influence how their learners perceive an MDA campaign. The study conducted in Zanzibar showed that teachers were less likely to accept the tablets until they were trained to teach about schistosomiasis.^10^

Teachers have an important role to play in MDA campaigns.^10,19^ This study indicates that many teachers working in rural schools had themselves been infected with schistosomiasis. Information about the current prevalence of schistosomiasis and the consequences of infection is required for both teachers and rural communities. Teachers are well placed to inform other adults and children in their communities.

## Conclusion

This study showed that teachers and learners know that swimming in rivers is a risk of contracting schistosomiasis, but they do not know why. It is important that people in schistosomiasis endemic areas be provided with the necessary information about the disease. It is neither complicated nor time-consuming to disseminate information about schistosomiasis and this study has shown how this essential information can be potentially implemented by teachers.
